# On the Discrepancy of Professional Opinions Respecting the Nature and Treatment of the Diseases Denominated, Generally, Venereal

**Published:** 1820-12

**Authors:** Richard Walker

**Affiliations:** Surgeon.


FOR THE LONDON MEDICAL AND PHYSICAL JOURNAL.

### *On the Discrepancy of Professional Opinions respecting the Nature and Treatment of the Diseases denominated, generally, Venereal.* By Mr. RICHARD WALKER, Surgeon.

IT is a law in mechanics, that when two forces, or powers, act in opposition to each other in an equal degree, the body, or subject, acted upon remains at rest; that is, its motion or effect is destroyed or suspended. By analogy it may likewise be inferred, that where two direct contrary opinions, of reputed equal credit or testimony, exist in the medical profession, such opinions completely invalidate each other, and leave the matter or question still equivocal.

This seems to be the case, to a most extraordinary degree, respecting the subject of the present observations. First, we have, from the most respectable authorities, two directly opposite opinions respecting the nature, and consequently the treatment, of the two very common affections of chancre and gonorrhœa virulenta. The opinion of Mr. HUNTER was, that those two affections were of the same nature, derived from the same origin, and propagated from the same source or infection; the difference in the two affections depending entirely upon the nature of the part it happened to be deposited on, as being a secreting or a non-secreting surface. Thus, if the matter happened to be deposited in the urethra, gonorrhœa was produced; if on the glans or prepuce, chancre. Moreover, that though, in his opinion, if the matter of gonorrhœa were absorbed into the system, it would produce the same secondary and constitutional affections as from chancre, it was, in this instance, ordinarily prevented by the secreting surfaces of the urethra; and, consequently, that gonorrhœa virulenta required, as well as chancre, for its cure and security against secondary affections, the same specific remedy—mercury.

The opinion of Mr. Benjamin Bell, a practitioner of probably equal authority in this matter, is the direct reverse of that of Mr. Hunter, viz. that the matter, or vitiated secretion, in gonorrhœa and in chancre, is essentially different in nature; that absorption of the matter of gonorrhœa, should it happen, will not produce secondary or constitutional affections; and that mercury is not necessary, as in chancre, either for the cure or prevention of secondary or constitutional affections.

The opinions of both these gentlemen have, at this time, each their abettors and followers, but mostly so on the side of the latter. Strange, however, as it may appear, the identity or non-identity of those two affections, respecting their specific nature, remain at this time unsettled, satisfactorily, by actual proof or demonstration.

Secondly, the opinion so long held universally in England, that mercury is a specific remedy, and the only one, for syphilis and its precursor, chancre, (excepting a late-proposed substitute for mercury, viz. nitric acid, or a mixture of nitric and muriatic acid, far from being generally adopted,) is now entirely exploded by some, particularly among one class of practitioners, gentlemen certainly possessing great opportunities of experience, but probably too liable to be led away by new doctrines and new practice.

These gentlemen contend that mercury may be entirely dispensed with as a specific or remedy in chancre and secondary affections of every description, and that these are safely curable, even without the use of any other specific, but by ordinary means alone; and they assert that the disease thus treated, although secondary symptoms may appear, likewise thus treated, will wear out, or exhaust itself: of which they give an apparently competent number of instances in proof of their assertion.

Thirdly, of late a new series of venereal affections has been discovered, differing entirely from genuine chancre and syphilis, and not requiring that specific, mercury, but which is aggravated by its use, denominated *pseudo-syphilis;* which till now, it should seem, and still is, by the ignorant, improperly treated with mercury, being confounded with genuine chancre and syphilis.

Fourthly, there are not wanting those who boldly assert that salivation by mercury, moderate or violent, however long continued or often repeated, is no remedy for the genuine disease.

Fifthly, that an affection called the *mercurial disease*, the effect of the injudicious use of mercury, resembles (like pseudo-syphilis) genuine syphilis so nearly as not to be, by some persons, distinguished from it; and the case of the unfortunate patient, of course, is materially injured by a perseverance in it.

Sixthly, some professional authors assert, confidently, that certain constitutional affections, not readily to be distinguished from genuine syphilis, are produced in consequence of sexual intercourse with a female so affected as to communicate a vitiated secretion, but not of a syphilitic nature. Others, of the first celebrity, go so far as to assert that constitutional affections, not readily, or with difficulty, to be distinguished from those, evidently occur in persons, from a naturally depraved or impure habit, without, or not arising from, sexual intercourse; and, of course, requiring a very different mode of treatment from those otherwise produced. And, lastly, there are not wanting those who assert, with equal confidence, that there is not, nor ever was, such an affection as the venereal disease!

To sum up the whole, there is no decided opinion, at present, respecting the *modus operandi* of mercury in curing syphilis; whether it operates, as some assert, by its occult specific property, or, as others say, by its chemical neutralizing quality, upon the disease.

All authors, however, seem to agree in the opinion, that venereal matter is, *cæteris paribus*, most virulent in the state of chancre; less so in bubo; and least so in the state of a venereal ulcer, the consequence of an affection of the system; and some go so far as to assert, that in this latter state it has quite, or nearly, lost its syphilitic character.

Amidst this diversity of clashing opinions, as above stated, actually existing at the present moment, it would be rendering the profession and the public no ordinary service to remove, even if it were in part only, these difficulties.

Having unavoidably, from the multifarious nature of the subject, been led into a longer and more tedious detail than I could have wished respecting the preliminary part of it, I shall endeavour to be as brief as possible in the part remaining.

Circumstances have of late years placed me much more in the practice of venereal cases than formerly, and sufficiently, I apprehend, to warrant my entering upon the subject with some tolerable share of confidence, but without the vanity of presuming that the observations I have to offer will tend much to elucidate this extremely difficult question in pathology.

With respect to the mode of treatment to be adopted in *gonorrhœa virulenta*, without going into the question whether that and chancre is or is not of the same specific nature, I am warranted in declaring, in conformity with the more commonly received opinion at this time, that that affection is curable, and safely so, without the use of mercury; having myself had experience of several hundreds of patients thus cured, and without the least consequent evil, though several years have since elapsed.

With respect to *genuine chancre*, distinctly characterized, I never trust to, or attempt, a cure without the use of mercury; nor entirely, in other venereal sores of every description that may occur, unless incidental circumstances appear to forbid it, or at least to warrant a suspension of it; and this, together with appropriate topical means, ordinarily of a mild, inoffensive nature, has never failed of producing the cure in a reasonable time, without any subsequent evil in either instance, whether the sore come under the description of genuine chancre or not.

I have had repeated experience of the ill effects from treating venereal sores on the genitals with caustic, escharotic, and strongly sedative, applications, especially in virulent cases, so as to check them too suddenly; bubo being almost the inevitable consequence: and, should this incidentally or incautiously happen, I, as speedily as I can, recal the former state of the chancre or venereal sore, being the lesser and more manageable evil of the two; the bubo ordinarily then disappearing.

With respect to *bubo*, I use the means ordinarily resorted to, according to circumstances, either for dispersion or suppuration; ever preferring, in the latter instance, opening it with the lancet rather than caustic, or suffering it to burst naturally; the after-treatment being thus more readily, effectively, and conveniently, managed.

*Venereal sore-throat.*—This I often find a very troublesome affection to manage. The first circumstance here is to ascertain correctly its origin, whether it be venereal or the effect of mercury. If there be any doubt respecting its appearance, and the patient has used much mercury, I suspend my opinion for a short time, directing an acidulous gargle, of muriatic acid or acetic acid properly diluted. I have commonly succeeded in curing it by this means, with the addition of some mild aperient and appropriate regimen: should it turn out otherwise, (known by its making progress, particularly with excavation,) I then use mercury, depending chiefly upon the unguent, in the same manner as when the syphilitic state of the ulcer or sore is, in the first instance, apparent; using a similar gargle, if necessary. Venereal sore-throat, like bubo, as before mentioned, often arises from a similar mismanagement of a chancre or venereal sore, by a kind of translation, common in venereal affections, from one part to another. Thus I have known a patient teazed for a long time, by the repeated alternations of sore on the genitals and sore in the throat; the virulent effects of the one being suspended during its action on the other part.

*Constitutional affections.*—The mode of treatment by a moderate use of mercury, administered for a primary affection, upon ordinary constitutions, so far precludes the occurrence of secondary symptoms, that a practitioner who has thus treated it need neither apprehend, nor is he likely to be reproached on account of, subsequent secondary or constitutional affections, provided his patient be of otherwise sound constitution.

When secondary symptoms, or constitutional, present themselves, evidently of syphilitic origin, mercury, I apprehend, judiciously managed, is the only, or the principal, remedy to be relied on.

*Anomalous affections.*—Under this head I would include all cases not assignable, or confined, to genuine syphilitic origin alone; whether it arise from a spurious source, which I have frequently met with in females, and communicated from them to men; or compounded of a genuine syphilitic affection grafted on scrofula, which occasionally occurs, and is a very perplexing case to manage.

*Pseudo-syphilis*, I consider as a kind of hybrid affection of the kind just mentioned, or the spurious; either of which, in certain constitutions, may be productive of very troublesome symptoms, exceedingly difficult, from their complicated nature, to conquer. Thus, I have occasionally met with cases in which syphilitic matter was grafted upon a scrofulous habit, where the sore of a bubo obstinately remained, after, I was well assured, all venereal taint was removed.

With respect to the notion that constitutional affections, so nearly resembling syphilis as not to be readily distinguished from them, without sexual intercourse, occur in certain depraved habits, from a fault in the chylopoietic functions, I consider it of a piece with the notion ludicrously broached formerly, of "Lucina sine concubitu."* It is to be lamented that the whole of the healing art comprehends too much for one individual to embrace completely, and that it is from a kind of necessity that it is ordinarily divided into the two branches of physic and surgery: from this cause, I am well assured, the practitioners of each branch separately, not possessing a competent view or knowledge of the whole collectively, arises that obscurity which is admitted in certain parts of the profession, and peculiarly so in the very intricate nature of the department of the profession which forms my present subject. Who is there that can determine precisely, in all cases, whether

* I should apprehend that no constitutional symptoms, resembling those in syphilis or pseudo-syphilis, can occur from the mere derangement or indisposition of the chylopoietic organs; but that such must ever arise from the absorption into the system of some poison or vitiated secretion, subject to different modifications, depending on peculiarity in the nature of the constitution or state of the system at the time.

mercury should be used or not, or to what extent it should be pursued, with the fullest or best advantage to the patient?

Syphilitic eruptions and sores, the effect of venereal intercourse, are, I apprehend, much less common now than formerly. We frequently meet with them in women, who, by repeated infections, or being constantly saturated, as it were, with syphilitic poison, have neglected proper treatment and care; but in men, who are only casually infected, even although they do not use the means, from neglect, apparently adequate to prevent such affections, yet they do not occur in them. These circumstances seem to prove that secondary or constitutional symptoms are not so necessarily the consequence of syphilitic primary symptoms, or that mercury is not so essential a preventive, as has been supposed.

I have frequently observed in buboes (after chancres) that have suppurated, opened with the lancet, and cured in moderate time, such person has afterwards, a sufficient length of time having elapsed, remained in perfect health; although he, by neglect, used scarcely any mercury. I am speaking now of persons of apparently pure constitutions, independently of the syphilitic affection. From all these considerations collectively, and the difference of opinion respecting the efficacy of mercury in genuine syphilitic affections, I have been induced to think less myself, of late, of its specific effect than formerly, and though not to dismiss it entirely, to use it with more moderation than I did some years since.

It is exceedingly difficult, as I have stated before, to distinguish, in some instances, the effects of mercury injudiciously employed, from the syphilitic affection itself, especially in the instance of sore-throat. Thus, I have witnessed an error occasionally from mistaking the ptyalism which is incident to a syphilitic sore-throat for the effects of mercury; and the ravages of the disease have been allowed to proceed under this mistaken impression. The fetor of the disease, and that from mercury, are palpably different: moreover, mercurial saliva will tarnish silver, whereas the syphilitic saliva will not; thus, it may readily be discovered when the latter has ceased and the former commenced.

From the obvious discordant and direct contrary opinions of professional men who possess the best opportunities of attending to this subject, which perhaps may be considered as a perfectly unique or detached part of the profession, and the acknowledged uncertainty and indecision in those men themselves respecting what cases are of a genuine syphilitic nature, and what are assignable to the nature of pseudo-syphilis, and consequently the dubious mode of treatment to be resorted to, I have considered it prudent in myself, and which may not be less so in other practitioners who may not possess the ample experience or leisure necessary for finally determining the various points of this most intricate subject, to be content with the following general maxims, as a basis for practice, which I would divide thus: First, such cases as are apparently of a syphilitic nature, in a habit otherwise apparently pure; secondly, such as occur in habits not pure, as scrofulous, scorbutic (so called), &c.; thirdly, such cases as appear to have been injudiciously treated with mercury, either in an excessive degree or too sparingly; and fourthly, such cases as are not in their nature apparently syphilitic; ever regarding these as somewhat suspicious, and regulating the treatment accordingly, by feeling as it were the effect of mercury upon them cautiously.

In the administration of mercury, whether externally or internally applied, or jointly, it should be steadily but cautiously persevered in, till the object for its use is completely effected. One course of mercury, however severe, is infinitely less injurious to the constitution than repetitions for the same complaint, considered as imperfectly treated before: it is this that deranges the constitution, disguises the real nature of the complaint, and puzzles the practitioner.

The topical treatment of the local affections incident to syphilis, as chancre, bubo, ulcerated throat, &c. is extremely important: dreadful must be the state of patients in many instances, if this be overlooked or injudiciously managed. The notion that local affections are to be cured by throwing mercury into the system, without the aid of topical applications, is a very dangerous one: the system may be duly secured by this means, whilst the local complaint is extending or getting worse, and this part of the treatment, especially in certain instances, requires great experience, with the utmost attention and retrospective reflection, to manage in the most efficient way.

The best plain rule for distinguishing syphilitic, from pseudo-syphilitic, affections, whether primary or secondary; where inspection or the history of the case is unsatisfactory, seems to be, the regular, uniform, progressive advance of the former, without irregularly shifting from one part to another, and the check to its further progress by the administration of mercury, and its ultimate cure; the reverse of which occurs in pseudo-syphilis.

The great difficulty in venereal cases, is to discriminate between what is syphilitic and what is not; and to distinguish what is peculiar to the disease itself, and what modifications of it arise from peculiarity of constitution, which are almost infinite; and, moreover, what may have arisen from maltreatment. This knowledge is to be obtained, principally, by attentive observation.

*Note.*—By the term *venereal*, I would be understood to mean all affections arising from sexual intercourse, divided into the specific distinctions of syphilitic and pseudo-syphilitic.

*Oxford; June* 30*th*, 1820.

